# Clinical Outcome of Ream Versus Unream Intramedullary Nailing for Femoral Shaft Fractures

**DOI:** 10.5812/ircmj.4631

**Published:** 2013-05-05

**Authors:** Farshid Bagheri, Seyed Reza Sharifi, Navid Reza Mirzadeh, Alireza Hootkani, Mohamad Hosein Ebrahimzadeh, Hami Ashraf

**Affiliations:** 1 Orthopedic and Trauma Research Center, Department of Orthopedic Surgery, Mashhad University of Medical Sciences, Mashhad, IR Iran

**Keywords:** Femur, Femoral Fractures, Fracture Fixation, Intramedullary

## Abstract

**Background:**

Stabilization of fractures with an intramedullary nail is a widespread technique in the treatment of femoral shaft fractures in adults; however, to ream or not to ream is still being debated.

**Objectives:**

The primary objective of this study was to determine clinical results following unreamed versus ream intramedullary nailing of femoral fractures.

**Patients and Methods:**

Between January 2008 and August 2009, 50 patients with femoral shaft fractures were treated with unreamed or reamed femoral nails in our clinic. From this prospective single centre study, 16 patients were excluded due to insufficient follow-up data. According to the AO classification, fractures in this study were either type A or B. Dynamic proximal locking was performed in all cases. The remaining 34 patients were divided into two groups of 17 with ream or unream nailing. During and after the operation, we evaluated some variables in whole series.

**Results:**

After statistical analyzes, we found that there were no differences in radiologic union time (P = 1) or full weight bearing time (P = 0.73) between ream and unream nailing. Nail breakage or iatrogenic fractures during nail insertion did not occur and we did not have any fat emboli in both groups but one secondary loss of reduction occurred in the unream group. Superficial infection after the operation was seen in one case which was treated successfully with antibiotics. In the ream group surgical time was about thirty minutes longer and differences were significant (P = 0.000). Patients had to pay more for ream nailing but the difference was not significant. We found no statistical difference between union time with or without reaming; on the other hand, there was significant increased operation length, blood loss and systemic changes in BP or So2 in the ream group versus the unream group.

**Conclusions:**

We advocate that unream nailing in traumatic femoral shaft fractures is a simple, safe and effective procedure with significant advantages, especially in multitrauma patients.

## 1. Introduction

Intramedullary nailing has become the standard treatment for diaphyseal femoral fractures. Proximal and distal locking of the intramedullary nail provides longitudinal and rotational stability (1, 2). In addition, nowadays, antegrade reamed femoral nailing is popular since it has a high union and low infection and malunion rate. However, several concerns have been raised regarding the local and systemic effects of reaming. Reaming disrupts the cortical blood flow and may cause variable degrees of thermal necrosis. With reaming procedures, the elevated intramedullary pressure can result in intravasation of fat and bone marrow contents. Reamed femoral nailing is associated with greater impairment of immune reactivity and with an increased consumption of coagulation factors. Intramedullary nailing also results in the stimulation of the inflammatory system. These systemic changes may contribute to pulmonary morbidity in patients with trauma (3-5).

To address these disadvantages of the reaming technique, solid nails with a smaller diameter were developed. Proponents of the unreamed nailing technique state that unreamed nails are faster to insert, specifically, less operation time, and have favorable results comparable to the reamed nails. But it couldn't be neglected that reaming provide better space, and makes it possible to larger nails to be applied. Thus, whether to ream or not is still debated (3, 6-8). In recent published review, it was concluded that reamed technique has better treatment results, we designed this study to compare clinical results of ream versus unream intramedullary nailing for closed femoral fractures (9).

## 2. Patients and Methods

Between January 2008 and August 2009, 50 patients with acute, traumatic femoral shaft fractures were treated by antegrade femoral nailing at Shahid Kamyab Trauma Center, Mashhad University of Medical Sciences, Mashhad, Iran. All patients were skeletally mature. Patients with a pathological fracture of the femur and patients who underwent secondary operations were excluded. Clinical records and radiographs were reviewed by authors. From this prospective single centre study, 16 patients were excluded due to insufficient follow-up data. We divided our patients in two groups (each group consists of seventeen patients with closed femoral shaft fractures). During and after surgery, we evaluated some variables and compared them between two groups. These factors included duration of surgery, bleeding during surgery, blood pressure change, O2 saturation change, cost of implant, radiologic union and the interval between surgery and full weight bearing. Blood loss during and after surgery was calculated by numbers of sponges, drainage collected in the suction system and hemovacs.

All patients were male with a mean age of 27 years (range 20–50 years) and all of the fractures were caused by a traffic accident. According to the AO classification, we selected patients with type A, or B fractures. All fractures were localized in the middle third of the femur. For unream cases we used a 9 mm solid nail (Synthes, Switzerland) and for ream cases a 11-13 mm nail (Synthes, Switzerland). All pateints in the study had isolated closed femoral fractures and we did closed intramedullary nailing (ream/unream) with C-arm control. The nails were dynamically locked. These patients were allowed progressive weight bearing in the first 6 weeks. There is no universally accepted definition of non-union; thus, we defined non-union as failure of clinical and radiological union after 9 months.

## 3. Results

Surgical time was about half an hour longer in the ream group, 118.2 minutes versus 79.2 minutes in the ream and unream groups respectively (P = 0.000). Bleeding during operation was averaged at 364.7 ml and 152.9 ml in the ream versus unream group respectively (P = 0.000). Change in blood pressure during reaming or insertion of the nail occurred in 11 patients in the ream group and in 4 patients in the unream group. This variable was also significant according to the x2 test (P = 0.015).

We also documented oxygen saturation changes during reaming or insertion of nails in both groups. According to this data, 6 patients of the ream group and only 1 patient of the unream group experienced SO2 changes (x2 test; P = 0.033). We did not find any significant difference between the two groups (ream/unream) in the case of full weight bearing interval (P = 0.7) and radiologic union (P = 0.1). In our country the cost of nails are higher for patients in the ream group than the unream group (P = 0.017). We did not have any fat emboli, implant failure and intra operative fracture. In one patient from the unream group (a type B fracture), loss of reduction occurred but he did not need revision surgery. Also, in one patient from the ream group, a superficial infection occurred that was treated with drainage and antibiotics successfully.

## 4. Discussion

Long bone fractures specially in femoral, are very common in orthopedic daily practice (10). According to a recent Swedish registry report, it's estimated that annual incidence of femoral shaft fracture is about 0.1% (11). Unfortunately we do not have a documented report of fracture frequency in Iran but we have recently a raise in traffic accident injured patients mostly with high velocity lower limb fractures in the country. The first locked intramedullary nail was introduced by Klemn and Shellam in 1972 and then developed by Kempf and Gross (12, 13).

Comparative studies of reamed and unreamed intramedullary nailing have given conflicting results, and most of them have included relatively small sample size of patients (14-16). Giannoudis et al. found no difference in the rate of non-unions in their studies. These authors recommended the use of an unreamed technique, as it is quicker to insert and performs as well as to the reamed technique (17). Several prospective, randomized clinical trials have been published comparing reamed and unreamed antegrade femoral nailing. The rate of non-union ranged from 1% to 2% in the reamed group and from 0% to 8% in the unreamed group (15, 16). Duan xin et al. report in their systematic review, significantly lower delay-union and non-union with the use of reamed nailing compared to undreamed nailing. (P = 0.002 and P = 0.02 respectively) (9).

Tornetta and Tiburzi analyzed 83 fractures that had reamed nailing and 89 fractures that had nailing without reaming. They found a significantly shorter time for union in the reamed group compared to the unreamed group. This was most evident in distal femoral fractures (18). This effect could be related to fracture debris in trauma site that provide an autologus bone graft (8). Selvakumar et al. randomized 102 consecutive patients with closed femoral shaft fractures into 2 groups, one reamed (n = 52) and the other unreamed (n = 50). They found that the rate of nonunion was 0% and 8%, respectively (19). Reaming of the femoral canal has been shown to increase intramedullary pressure, including intravasation of bone marrow and fat into the venous blood system (20, 21).

Elevated pressure can result in fat embolism syndrome (FES), acute respiratory distress syndrome (ARDS), and even sudden death. In our study with unreamed femoral nailing there were no cases of FES or ARDS. To what extent reaming increases the risk for pulmonary complications is still unclear. Pape et al. have suggested that reaming the femoral canal may have a detrimental effect on pulmonary function and recommended nailing without reaming to reduce the risk of ARDS (22). Buckley et al. reported in a prospective, randomized study of 153 patients with isolated femoral fractures that there was no difference in pulmonary complications for reamed vs. unreamed intramedullary nails (23). In a large prospective, randomized, multicentre study, the Canadian Orthopedic Trauma Society found no significant difference in ARDS, between the reamed and unreamed groups. They also reported that the ARDS rate was too low to detect a significant difference (6). Xin Duan’s study also, did not detect significant (P = 0.78) difference in his review (9).

Bone et al. confirmed these findings and recommended that patients with pulmonary injuries and femoral fractures should have reamed intramedullary stabilization unless they are hemodynamically unstable. In these latter cases, they recommended early stabilization, but with use of an unreamed nail or plating technique (24). In this study, the incidence of infection in the ream group was low. We only had one case of superficial infection and without any deep infection, which is comparable with other studies. The reported incidence of infection complicating reamed intramedullary nailing varies between 0% and 3.3% (14). The infection rate in patients treated with unreamed nailing ranges from 0% to 2.9% (2, 14). However, some studies, reported no significant difference in infection rate between reamed and unreamed nailing (7, 17).

In the current study blood loss was higher in the ream group than the unream group (P = 0.00). Tornetta IIIP and Tiburzi D found similar results with more blood loss in the ream group (18). Surgical time was about half an hour longer in the ream group and in comparison to the unream group this difference was statistically valuable (P = 0.00). In our study we had no nail breakage; one secondary loss of reduction occurred in the unream group but angulations were acceptable and did not need revision. There was no statistically different in implant failure rates in recently published review, too (9).

Blood pressure and oxygen saturation during the operation were compared between ream and unream groups which showed statistical differences (P = 0.015 for BP change and P = 0.033 for oxygen saturation difference). A weakness in our study was that our series was small, but we think our results are reasonable. We advocate that unream nailing in traumatic femoral shaft fractures is a safe and effective procedure, especially in multi-trauma patients. The debate of whether to ream or not still continues. A large multicentre, randomized, controlled trial with sound methodology is needed to make a solid recommendation.
